# The Role of Galectin-3 in the Kidneys

**DOI:** 10.3390/ijms17040565

**Published:** 2016-04-14

**Authors:** Szu-Chia Chen, Po-Lin Kuo

**Affiliations:** 1 Graduate Institute of Clinical Medicine, College of Medicine, Kaohsiung Medical University, Kaohsiung 807, Taiwan; scarchenone@yahoo.com.tw; 2 Division of Nephrology, Department of Internal Medicine, Kaohsiung Medical University Hospital, Kaohsiung Medical University, Kaohsiung 807, Taiwan; 3 Department of Internal Medicine, Kaohsiung Municipal Hsiao-Kang Hospital, Kaohsiung Medical University, Kaohsiung 812, Taiwan; 4 Faculty of Medicine, College of Medicine, Kaohsiung Medical University, Kaohsiung 807, Taiwan; 5 Institute of Medical Science and Technology, National Sun Yat-Sen University, Kaohsiung 804, Taiwan

**Keywords:** galectin-3, kidney, clinical renal disease, nephrogenesis

## Abstract

Galectin-3 is a 32- to 35-kDa member of the galectin family of b-galactoside-binding lectins, which is characterized by a carbohydrate recognition domain. Through its carbohydrate-binding function, it regulates cell growth, differentiation, and inflammation. It also plays a complex, context-dependent role in the kidneys. During development, it promotes nephrogenesis and is strongly expressed in the ureteric bud and its derivatives. An increase in the concentration of galectin-3 has been reported to be associated with fibrosis of the kidneys. Elevated levels of plasma galectin-3 are also associated with increased risks of rapid renal function decline, incident chronic kidney disease, and progressive renal impairment, and also with cardiovascular end points, infection, and all-cause mortality in patients with renal function impairment. This review discusses a general survey on galectin-3 expressions in nephrogenesis, kidney injury animal models, clinical renal diseases, renal transplantation and the potential role of galectin-3 for treatment in kidney disease.

## 1. Introduction

Galectins are a group of proteins that can bind to β-galactoside sugars by either N-linked or O-linked glycosylation through their carbohydrate recognition domain [1,2,3]. First isolated in 1976, there are now 15 different galectins that have been characterized and they are numbered according to their order of discovery (galectin-1 to galectin-15). They are also widely distributed from lower to higher vertebrates [1,2].

To date, galectin-3 is the only one discovered in mammals [1,2]. It is a 32- to 35-kDa multi-functional lectin protein expressed by epithelial and endothelial cells, and macrophages. Galectin-3 regulates numerous biological processes through its carbohydrate recognition domain using carbohydrate-independent mechanisms [4,5]. Predominantly located in the cytoplasm, galectin-3 can be secreted extracellularly but can also shuttle into the nucleus. Extracellular galectin-3 modulates important interactions between epithelial cells and extracellular matrix, and plays a role in the embryonic development of collecting ducts [6]. In contrast, intracellular galectin-3 is important for cell survival due to its ability to block the intrinsic apoptotic pathway, while intra-nuclear galectin-3 promotes cell proliferation [4,5].

**Figure 1:**
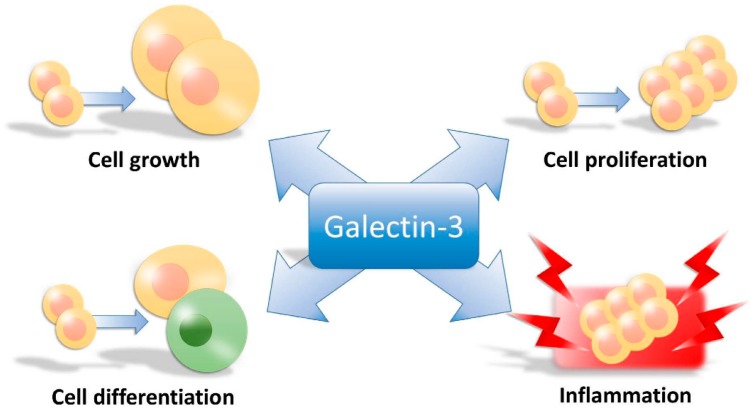
The action of galectin-3 in the cells.

There are several known ligands, including various glycosylated matrix proteins (*i.e.*, laminin, fibronectin, and integrins), for galectin-3 [7]. Cell adhesion and proliferation due to galectin-3 may translate into pathological processes like fibrosis and cancer progression. In a series of clinical and experimental evidences, galectin-3 has been involved in fibrosis, heart failure, obesity, impaired glucose metabolism, and cancer [8,9,10,11,12]. Galectin-3 has also been implicated in the pathogenesis of ventricular remodeling, infections, and various autoimmune and inflammatory processes [9,13]. In acute tissue damage, for instance, it is a key component in the host defense against microbes such as Streptococcus pneumonia [14]. Therefore, galectin-3 regulates cell growth, proliferation, differentiation, and inflammation (Figure 1).

However, in repetitive tissue injury, galectin-3 also appears to be closely involved in the development of chronic inflammation, helping wall off the tissue injury via fibrogenesis and organ scarring [9]. As such, galectin-3 may be regarded as a regulatory molecule affecting various stages from acute to chronic inflammation and tissue fibrogenesis.

The current review aims to present a general survey on galectin-3 expression in nephrogenesis, kidney injury in animal models, clinical renal diseases, and renal transplantation, as well as its potential role in the treatment of kidney disease.

## 2. The Role of Galectin-3 in Nephrogenesis

Galectin 3 can be found by immunofluorescence in principal and intercalated cells of collecting duct of the kidneys, as well as in the thick ascending limbs at lower levels [15]. During metanephros, the adult kidney precursor, galectin-3 can be detected in the apical domains of ureteric bud branches. It is also intensely expressed in fetal medullary and papillary collecting ducts of both cytoplasmic and plasma membranes. Low levels of galectin-3 are likewise found in the cytoplasm of a subset of cells in adult collecting ducts [16]. Galectin-3 can modulate branching morphogenesis of the mouse ureteric bud/collecting duct lineage, but is not detected in early metanephrogenesis. Later, it is upregulated in fetal kidney maturation and prominent in the basal domains of medullary collecting ducts [17].

Galectin-3 expression is also reported in the later stages of nephrogenesis, when its expression is confined to ureteric bud derivatives like the collecting ducts and connecting segments of distal tubules [6,18]. In normal adult kidneys, it is restricted to the collecting tubules and primary cilium, aside from being temporarily expressed by altered proximal tubules during regeneration [19,20].

## 3. Presentation of Galctin-3 in Animal Models of Renal Failure

Nishiyama *et al.* [19] evaluated the association of galectin-3 with cell injury and regeneration in ischemic and toxic acute renal failure (ARF). Galectin-3 mRNA began to increase at 2 h and increased 6.2-fold at 48 h before decreasing 28 days after the injury. By immunohistochemistry, galectin-3 began to develop in the proximal convoluted tubules 2 h after reperfusion. From 6 to 48 h, the authors also observed galectin-3 in proximal straight and distal tubules, thick ascending limbs, and collecting ducts, and then in macrophages during the later stages of regeneration. Thus, the authors concluded that galectin-3 expressions were markedly up-regulated in both ischemic and toxic types of ARF, suggesting that it might play an important role in acute tubular injury and the subsequent regeneration [19].

Macrophages are posited to be a key cell type in the pathogenesis of renal fibrosis [21]. Galectin-3 is up-regulated in a mouse model of progressive renal fibrosis (unilateral ureteric obstruction). Its absence is protective against renal myofibroblast accumulation and activation, and fibrosis, but its secretion by macrophages is essential to the activation of renal fibroblasts to a profibrotic phenotype [22]. In a study of galectin-3 in progressive fibrosis, galectin-3 not only protected the renal tubules from chronic injury by limiting apoptosis, but also led to enhanced matrix remodeling and the attenuation of fibrosis [23].

## 4. Galctin-3 and Advanced Glycation End-Products (AGEs) in Animal Models of Diabetic Nephropathy

Pugliese’s study on the role of galectin-3 and its contribution to the development of diabetic glomerular disease aimed to evaluate the *in vivo* role of galectin-3 and its functional role in facilitating the removal of AGEs and/or mediating the effects of these adducts in terms of cell activation and tissue injury induction [24]. The authors reported that the mice deficient in galectin-3 developed glomerulopathy with a more pronounced increase in proteinuria, expression of the extracellular matrix gene, and expansion of mesangial cells, all of which were associated with higher renal/glomerular AGE accumulation. In turn, this was associated with the absence of functioning galectin-3 AGE receptors. Taken together, these suggested that galectin-3/AGE-receptor 3-deficient mice developed diabetic glomerulopathy faster [24].

Another study on the role of galectin-3/AGE-receptor function in the pathogenesis of diabetic renal disease revealed that galectin-3 knockout mice had higher circulating and renal AGE levels, and exhibited more marked renal functional and structural changes after injection of *N*-carboxymethyllysine-modified or unmodified mouse serum albumin [18]. Functionally, the mice had significantly high proteinuria and albuminuria. Besides, morphological evaluation of kidney from galectin-3 knockout mice showed significant glomerular sclerosis, with periodic acid Schiff positive deposits within the mesangium and thickening of glomerular basement membrane and Bowman’s capsule. The association between a reduction in galectin-3 and greater susceptibility to AGE-induced renal disease, increased levels of AGE and signaling, and altered patterns of AGE-receptors all suggest that galectin-3 functions as an AGE receptor *in vivo*, thereby providing protection against AGE-dependent tissue injury [18].

## 5. Galctin-3 Presentation in Animal Models of Ischemia/Reperfusion Injury (IRI)

In Vansthertem’s study on renal regeneration, galectins and their binding sites in rat kidneys were detected by histochemistry after ischemic injury. After ischemia, a population of galectin-3 (+), CD44 (+), and vimentin (+) interstitial round cells located in the outer stripe of the outer medulla were detected around the necrotic tubules and in the lumen of adjacent blood vessels. These suggested that galectin-3 might be involved in the complex process of post-IRI kidney regeneration [25].

The study of Fernandes Bertocchi evaluated the role of galectin-3 in IRI-triggered inflammation. Galectin-3 knockout animals presented with less acute tubular necrosis and more prominent tubular regeneration compared to controls, and had lower expressions of monocyte chemotactic protein 1, interleukin-6, and interleukin-1β, less macrophage infiltration, and lower reactive oxygen species production early in the disease process [26]. Galectin-3 seemed to play a role in renal IRI, especially in the secretion of macrophage-related chemokines and pro-inflammatory cytokines, and in the production of reactive oxygen species.

## 6. Galectin-3 in Human Diabetic Nephropathy

In animal models of diabetic nephropathy or ARF, galectin-3 has been shown to be upregulated [19,22,24]. Kikuchi *et al.* [27] investigated the expression of galectin-3 in renal biopsy specimens from patients with diabetic, membranous and IgA nephropathy, crescentic glomerulonephritis, and minimal change nephrotic syndrome. In normal human kidney, galectin-3 was found in the distal tubules but not in the glomeruli. Moreover, there were significantly more galectin-3-positive cells in the glomeruli of diabetic nephropathy than in the glomeruli of other nephropathies. The ratio of galectin-3-positive cells to the total number of macrophages in the tubules was also significantly increased in diabetic nephropathy.

In diabetic patients, there was a significant correlation between the number of galectin-3-positive cells in the glomeruli and urinary protein excretion, but a negative correlation between the number of galectin-3-positive cells in the glomeruli and the regression rate of renal function [27]. These findings suggest that the infiltration of galectin-3-positive cells may play an important role in the progression of diabetic nephropathy such that the degree of galectin-3 expression may be a predictor of poor prognosis.

## 7. Galectin-3 in Systemic Lupus Erythematosus (SLE) Nephritis

Kang *et al.* [28] examined 88 patients with SLE nephritis and five normal specimens for galectin-3 expression patterns in renal tissues of patients with SLE nephritis to determine whether tissue and serum galectin-3 were associated with SLE nephritis. Glomerular galectin-3 expression was noted in 81.8% (72/88) of patients with SLE nephritis but not in the five controls. The galectin-3 expression levels correlated with histologic activity indexes, anti-dsDNA titers, and levels of complements 3 and 4. Serum galectin-3 levels were higher in patients with SLE, especially in those with nephritis, and correlated with anti-dsDNA titers. Patients with SLE nephritis had higher serum galectin-3 levels and glomerular galectin-3 expression in renal tissue, which reflected disease activity. These findings suggest that galectin-3 may contribute to the inflammatory process in SLE.

## 8. Elevated Galectin-3 in Chronic Heart Failure

Galectin-3 is secreted by activated macrophages and is implicated in the regulation of cardiac pro-inflammatory and pro-fibrotic pathways [29,30]. In rodent models, myocardial galectin-3 expression can predict future heart failure [31], while the administration of exogenous galectin-3 can promote fibrosis and heart failure [29]. On the other hand, genetic or pharmacologic inhibition of galectin-3 attenuates fibrosis and cardiac dysfunction in response to pro-fibrotic stimuli [30,32].

Plasma galectin-3 was elevated in patients with chronic heart failure and high plasma galectin-3 level was associated with renal insufficiency [33]. Plasma galectin-3 is also a novel prognostic marker for mortality in chronic heart failure and its prognostic value is independent of the severity of heart failure [8,34,35]. The link between renal insufficiency, heart failure and galectin-3 is not completely understood. Gopal *et al.* [36] evaluated whether the relationship between renal function and galectin-3 was influenced by clinical decompensation, type of heart failure, or the presence or absence of clinical heart failure. They found galectin-3 was inversely related to renal function regardless of having heart failure. Concentrations of galectin-3 did not seem to depend on the level of compensation or type of heart failure. Furthermore, the value of glaectin-3 for heart failure prognosis declined after adjusting for renal function [35]. These findings raised an important consideration that renal impairment is a major determinant of galectin-3 in patients with heart failure [35,36]. Despite markedly increased plasma galectin-3 levels in patients with heart failure, their urine galectin-3 levels are not increased. Impaired renal handling of galectin-3 in such patients may explain the relationship between renal function and galectin-3, and may account for the elevated plasma galectin-3 in heart failure [37].

## 9. Galectin-3 Is Associated with Renal Outcomes in Chronic Kidney Disease (CKD)

CKD is a major worldwide public health concern as it can lead to progressive kidney function deterioration, substantial morbidity, and increased mortality via cardiovascular and non-cardiovascular causes [38,39,40,41,42]. Nonetheless, the decline in kidney function may be slowed or even reversed by early detection, thereby avoiding secondary complications [43]. Thus, it is important to find novel biomarkers that can identify at-risk individuals at the earliest possible stage. Galectin-3 has been linked to the development of renal fibrosis in animal models [22] and is inversely correlated with estimated glomerular filtration rate (eGFR) in humans [33]. However, whether or not it can predict kidney disease remains unknown.

Conall *et al.* [44] assessed renal outcomes for 2450 Framingham Offspring participants during a mean follow-up of 10.1 years. The eGFR rapidly declined (≥3 mL/min/1.73 m^2^/year) in 241 (9.2%) participants, while incident CKD (eGFR < 60 mL/min/1.73 m^2^) and albuminuria (albumin-to-creatinine ratio ≥17 mg/g in men; ≥25 mg/g in women) developed in 277 (11.3%) and in 194 (10.1%), respectively. They also found an association between higher levels of plasma galectin-3 and a rapid decline in eGFR as well as a higher risk of incident CKD but not incident albuminuria.

Subclinical tubule-interstitial fibrosis may be important in the early stages of CKD, while the lack of association with albuminuria contradicts the hypothesis that glomerular injury or foot process effacement is a mechanism. Higher circulating levels of galectin-3 have been associated with a higher risk of incident CKD and decline in renal function, suggesting that levels of galectin-3 can be used to predict kidney injury years before CKD is detected clinically, allowing for the early provision of targeted treatment and disease prevention. However, the number of serum creatinine measurements for making the eGFR decline was twice. Therefore, renal function change could not be observed well. Besides, they used the baseline galectin-3 for analysis. Data of time-dependent changes in galectin-3 were not obtained, and they were not able to investigate the association between galectin variation and outcomes.

## 10. Galectin-3 Is Associated with Clinical Outcomes in CKD

In renal disease, galectin-3 can play a role in the onset and development of diabetic and non-diabetic nephropathy [45]. Using a model of unilateral ureter obstruction, a recent study demonstrated that genetic disruption of the gene encoding galectin-3 attenuated renal fibrosis [22]. However, associations between galectin-3, renal function, and adverse outcomes have not been reported in the literature. Drechsler *et al.* [46] analyzed two large groups of patients with a wide variation in renal function from the German Diabetes Mellitus Dialysis (4D) study (1168 patients with type 2 DM undergoing dialysis) and Ludwigshafen Risk and Cardiovascular Health (LURIC) study (2579 patients who underwent coronary angiography). Galectin-3 concentrations were measured at baseline, and the patients were stratified into three groups: eGFR ≥ 90, 60–89, and <60 mL/min per 1.73 m^2^. The results showed that galectin-3 was not associated with long-term outcomes in the ≥90 mL/min per 1.73 m^2^ group in the LURIC study [46], but that it was significantly associated with all-cause mortality, cardiovascular mortality, death due to infection, and sudden cardiac death in patients with eGFR of 60–89 mL/min per 1.73 m^2^. In patients with an eGFR <60 mL/min per 1.73 m^2^, galectin-3 concentration was also associated with myocardial infarction and death due to chronic heart failure. In the 4D study, the level of galectin-3 was found to predict all-cause mortality, cardiovascular events, stroke, and mortality due to an infection [46].

In previous studies, increased galectin-3 levels were associated with adverse clinical outcomes in the general population and in patients with heart failure [47,48]. The study by Drechsler *et al.* [45] extended this association to patients with renal disease. However, this study utilized a *post hoc* analysis within two selected cohorts of German patients. Therefore, the generality of the relationship between elevated galectin-3 and adverse outcomes was limited. In addition, proteinuria amount was not measured, which was an important variable influencing renal and cardiovascular outcomes.

## 11. Galectin-3 Is Associated with Renal Transplantation

Although renal transplantation (RTx) has dramatically improved patient survival and short-term quality of life, long-term allograft survival is still a major challenge [49]. The mechanisms leading to late graft loss are not well known and the risk factors in kidney transplant recipients warrant further elucidation. Thus, it is imperative to identify biological markers associated with poor transplant outcomes for the development of therapeutic strategies to improve long-term success.

Previous studies have suggested that increased galectin-3 may be an important biomarker for renal disease [22,45]. However, the galectin-3 expression patterns in RTx patients are not yet known. The study by Tan [50] investigated serum galectin-3 levels in hemodialysis and RTx recipients. The study included 41 normal subjects, 41 RTx recipients, and 32 hemodialysis patients. Galectin-3 levels in the RTx and hemodialysis groups were higher than in normal subjects. In paired analyses, galectin-3 levels were significantly decreased in RTx patients at 3 months, did not change in the hemodialysis group, and high in the maintenance hemodialysis patients. Kidney transplantation improved galectin-3 levels. However, in this study, the limitation is the unknown effect of immunosuppressive drugs and other drugs (e.g., aspirin, statins) on galectin-3 expression.

Furthermore, to examine the role of galectin-3 in chronic allograft injury (CAI), Dang *et al.* [51] adopted a murine model of CAI characterized by a single class II mismatch between BM12 donor and C57BL/6 recipient strains. The transplantation of BM12 kidneys into C57BL6 mice was associated with interstitial fibrosis, tubular atrophy, and the up-regulation in galectin-3 expression. In contrast, the transplantation of BM12 kidneys into galectin-3 null mice led to a significant preservation of tubules, reduced interstitial fibrosis, and decreased myofibroblast activation and collagen I expression. Thus, galectin-3 may promote renal transplant fibrosis, suggesting a potentially exciting therapeutic target in CAI.

## 12. Galectin-3 as a New Treatment Opportunity for Renal Disease

**Figure 2:**
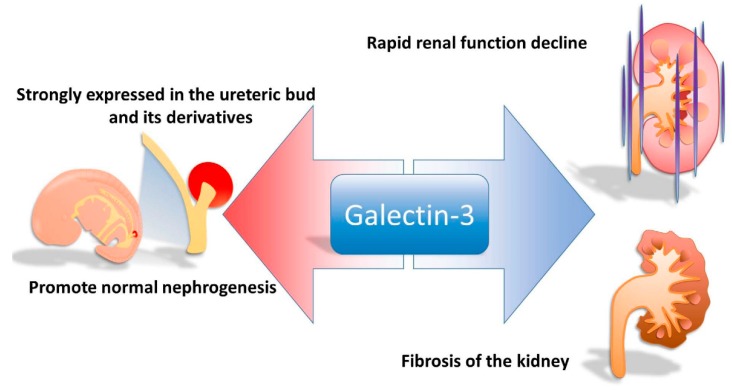
The influence of galectin-3 in the kidneys.

Galectin-3 has been reported to promote nephrogenesis and to be strongly expressed in the ureteric bud and its derivatives. Furthermore, an increased concentration of galectin-3 has been reported to be associated with fibrosis of the kidneys. Furthermore, elevated levels of plasma galectin-3 are associated with increased risks of rapid renal function decline and of incident chronic kidney disease (Figure 2). Galectin-3 has also been reported to play a role in ameliorating inflammation, with an increased concentration in response to ischemia and nephrotoxic acute kidney injury [19]. In addition, it has also been reported to prevent chronic tubular injury by reducing apoptosis and fibrosis, and increasing matrix remodeling [23]. Its reno-protective properties are evidenced by the rapid development of diabetic nephropathy in galectin-3^–/–^ mice [24]. However, in persistent or repetitive tissue injury, it may modulate the transition to chronic inflammation and fibrosis [9].

Galectin-3 is a potent activator of fibroblasts in the kidneys [52]. Although kidney fibrosis in human transplant recipients depends on galectin-3 [51], the role of elevated galectin-3 levels in providing more benefits from specific therapy warrants further studies. Sun *et al.* [53] investigated the effects of AGE and rosiglitazone on galectin-3 expression and secretion in cultured human renal mesangial cells. Rosiglitazone increased galectin-3 expression and secretion in a dose-dependent manner, suggesting that rosiglitazone may play a role in reno-protection via galectin-3 up-regulation.

Although galectin-3 is up-regulated in acute kidney injury, the relative importance of its different domains and functions are poorly understood. Kolatsi-Joannou *et al.* [54] investigated changes in the expression of galectin-3 in mice with acute kidney injury induced by folic acid using modified citrus pectin (MCP), a pectin derivative which can bind to the galectin-3 carbohydrate recognition domain. This antagonized functions linked to this role. The MCP reduced renal cell proliferation but did not affect apoptosis. At two weeks of the recovery phase, MCP-treated mice demonstrated reduced galectin-3, with decreased renal fibrosis, macrophages, pro-inflammatory cytokine expression, and apoptosis. Their findings reveal that MCP was protective in experimental nephropathy by modulating early proliferation and later, galectin-3 expression, apoptosis, and fibrosis. Thus, MCP may be a novel target to reduce long-term renal injuries, possibly via the effect of galectin-3 on carbohydrate binding-related functions.

A recent phase II study comparing treatment with a galectin-3 inhibitor (GCS-100) and a placebo in patients with CKD stage 3b showed that GCS-100 significantly improved the eGFR [55]. Taken together, galectin-3 may be used to assess the prognosis, guide therapy, and potentially suggest specific anti-galectin-3 therapy. However, prospective studies are needed to validate these hypotheses.
